# The LMC Skills, Confidence & Preparedness Index (SCPI): development and evaluation of a novel tool for assessing self-management in patients with diabetes

**DOI:** 10.1186/s12955-017-0606-z

**Published:** 2017-01-31

**Authors:** Lawrence Mbuagbaw, Ronnie Aronson, Ashleigh Walker, Ruth E. Brown, Naomi Orzech

**Affiliations:** 1 Department of Clinical Epidemiology and Biostatistics, McMaster University, Hamilton, ON Canada; 2 Biostatistics Unit, Father Sean O’Sullivan Research Centre, St Joseph’s Healthcare, Hamilton, ON Canada; 3 Centre for the Development of Best Practices in Health, Yaoundé Central Hospital, Yaoundé, Cameroon; 4 LMC Diabetes & Endocrinology, 1929 Bayview Ave., Toronto, ON M4G 3E8 Canada

**Keywords:** Diabetes, Self-efficacy, Measurement, Self-management, Patient education

## Abstract

**Background:**

Optimal diabetes care requires a specific set of self-management behaviours. The purpose of this study was to present the development and initial psychometric evaluation of a new tool to measure three key aspects of a patient’s diabetes self-management: knowledge of the skill, confidence in being able to perform the skill and preparedness to implement the skill.

**Methods:**

A sequential exploratory mixed-methods design was used. A panel of educators, researchers and clinicians established a scale with items that would adequately capture skills, confidence and preparedness in seven core health behaviours central to diabetes care. The psychometric properties of the items were pilot tested on 120 participants with diabetes from a tertiary referral centre, and repeated 6 months later on 70 participants. Item selection was informed by factor analysis, item-total statistics and the need for brevity.

**Results:**

Twenty five items from a pool of 36 were retained, with an excellent overall intraclass correlation (ICC) of 0.94 (95% CI 0.92–0.99; *p* < 0.001). Internal consistency for the subscales (skills-9 items, confidence - 8 items, preparedness – 8 items) was very good (intraclass correlation between 0.83 and 0.88), and retest reliability after 6 months was also good (*r* = 0.48; *p* < 0.01). The scale was positively correlated to established scales that assess skill (Michigan Diabetes Knowledge Test) (*r* = 0.21;*p* = 0.01), and assess skill and confidence (Diabetes Empowerment Scale) (*r* = 0.28;*p* < 0.01).

**Conclusions:**

The Skills, Confidence & Preparedness Index is a brief and easy to administer new scale that is more comprehensive than existing tools. It should be used to assess self-management in patients with diabetes, optimize the resources applied to each patient, and determine educational needs and direct clinical management. The scale should be further evaluated in a broader population of patients with diabetes.

**Electronic supplementary material:**

The online version of this article (doi:10.1186/s12955-017-0606-z) contains supplementary material, which is available to authorized users.

## Background

Diabetes is a chronic disease affecting more than 300 million people world-wide [1] and contributing to the global burden of complications such as blindness, chronic kidney disease, and amputation. The multiple parallel interventions necessary for optimal care require both a multidisciplinary approach to care and a specific set of self-management skills. Patients are tasked with frequent daily decisions about their lifestyle, medication and therapies that must be effective and yet align with their lifestyles, while accommodating many physiological and psychosocial factors. Therefore, it is critical that they are knowledgeable about these skills, confident that they are capable of making the required change and prepared to actually implement the behaviour. Diabetes education, in particular promoting effective self-management behaviours, is considered a critical aspect of diabetes care [2].

Many tools have been developed to measure patient-level indicators of diabetes care, some of which are summarised in Table 1 [3–22]. Although tools are currently available to specifically assess the knowledge [9, 11] or self-efficacy [3, 12] of diabetes self-management, most of the available tools are unidimensional, and none are sufficiently comprehensive to capture the multidimensional components of diabetes self-management. The authors recently published the results of DROP A1C – an education program that used the LMC Barriers to Care Questionnaire to evaluate barriers to glycemic control in patients with persistently uncontrolled glycemia (refractory patients) [23]. Patients expressed a wide range of different types of barriers, such as a lack of diabetes education/knowledge, fear, anxiety, and lack of motivation. It became clear that in order for educators to effectively tailor a patient’s education to their individual needs, a comprehensive tool was needed that could capture not only a patient’s knowledge of diabetes self-management, but also their confidence in their diabetes management skills, and how prepared they felt to implement behavioural changes. Thus, a working group composed of national experts in diabetes care was formed to devise a series of questions that would optimally assess each of three dimensions that contribute to diabetes self-management: patients’ knowledge of the skill; their confidence in being able to perform the skill; and their preparedness to actually apply the skill to self-manage their diabetes. Each dimension was determined to be a necessary component for successful diabetes self-management, and no current tool is available to assess all three dimensions. The working group prioritized development of a questionnaire that could be easily implemented in clinical practice to optimally tailor specific education to individual patient’s needs. The development of the resulting LMC Skills, Confidence & Preparedness Index (SCPI) and its first psychometric evaluation is reported here.

**Table 1:** Summary of diabetes assessment tools

Name of tool	Aspect of care assessed	Number of items
Problem areas in diabetes (PAID) [18]	Diabetes specific emotional distress	20
Diabetes Treatment Satisfaction Questionnaire (DTSQ) [6]	Treatment satisfaction	8
Audit of Diabetes-Dependent Quality of Life (ADDQoL) [7]	Impact of diabetes and its treatment on quality of life	13
Appraisal of Diabetes Scale (ADS) [8]	Individuals appraisal of diabetes and how it affects their life	7
Diabetes Care Profile (DCP) [10]	Social and psychological factors associated with Diabetes and it’s treatment	234
Diabetes-39 Questionnaire (D-39) [5]	Quality of life in diabetic patients	39
Diabetes Health Profile (DHP) [17]	Eating, activity and psychological distress	32
Diabetes Impact Measurement Scales (DIMS) [14]	Symptoms, well-being, moral and social life	44
Diabetes Quality of Life Clinical Trial Questionnaire (DQLCTQ) [20]	Changes on quality of life for diabetic patients in clinical trials	142
Diabetes Quality of Life Measure (DQOL) [13]	Life satisfaction, diabetes impact, worries about diabetes and social concerns	46
Diabetes Specific Quality-of-Life Scale (DSQOLS) [4]	Treatment goals, burden of diabetes care and management	64
Questionnaire on Stress in Patients with Diabetes – Revised (QSD-R) [15]	Treatment goals, treatment success and burden of diabetes care and management	64
Well-being Enquiry for Diabetics (WED) [16]	Quality of life	50
Diabetes Empowerment Scale (DES) [3]	Psychosocial self-efficacy	37
Diabetes Knowledge Test (DKT) [9]	General Knowledge of Diabetes	23
Diabetes Self-Efficacy Scale [12]	Self-efficacy of diabetes self-care	12
Diabetes Self-Management Questionnaire (DSMQ) [19]	Diabetes-specific self-care activities associated with glycemic control	16
Diabetes Knowledge Questionnaire (DKQ) [11]	General Knowledge of Diabetes	24
Confidence in Diabetes Self-Care Scale (CIDS) [22]	Confidence in diabetes-specific self-care behaviours	20
Summary of Diabetes Self-Care Activities Measure (SDSCA) [21]	Activities associated with diabetes self-management	25

## Methods

### Instrument development

A sequential exploratory mixed-methods design was used to develop the tool [24]. In the first qualitative phase, items were developed for appraisal. In the second quantitative phase, these items were tested on participants using structured questionnaires.

A working group of experts in diabetes care from multidisciplinary fields (endocrinology, dietetics, and nursing), with contributions from psychiatry and primary care, began developing the tool in November 2013. The goal was to develop a tool that assessed various aspects of self-management in patients with type 1 and type 2 diabetes, and that could be easily used in clinical practice.

The multidisciplinary expert panel convened over monthly in-person meetings and teleconferences. Prior to the development of the questionnaire, the panel completed a needs assessment that was guided by collective clinical experience, including questions previously explored in prior research in refractory patients, [23, 25] and by the Canadian Clinical Practice Guidelines (CPG) [26]. Currently published and validated diabetes assessment tools in the context of patient knowledge and self-efficacy, such as The Michigan Knowledge Test [9], The Diabetes Empowerment Scale [3], and the LMC Barriers to Care Questionnaire [23] were reviewed. The panel also decided to incorporate the American Association of Diabetes Educators Self Care Behaviors (AADE7 Self-Care Behaviors™) [27].

Based on the needs assessment, the expert panel confirmed the importance of the assessment of self-management skills [2], and first developed 15 items that evaluated diabetes self-management skills. The content for the items was mainly guided by the CPG and the AADE7 Self-Care Behaviours, and included key areas of focus for diabetes self-management, including healthy eating, being active, blood glucose monitoring, medications, problem solving, reducing risk and healthy coping [27]. The panel then used the framework from the Social Cognitive Theory [28] to create 15 items that assessed the patients self-efficacy of performing those diabetes self-management skills. Self-efficacy, defined as a person’s beliefs or confidence about their abilities to perform a skill, is associated with better self-management behaviours, and more optimal glycemic control [29]. The expert panel continuously appraised each item for clarity and conciseness, and eliminated 3 skills questions and 3 confidence questions after a consensus that the questions were redundant. Finally, the panel developed 12 more items that specifically addressed how prepared a patient was to implement self-management behaviours, reflecting on the Transtheoretical Model of Health Behaviour Change [30]. The diabetes educators were well familiar with this model and already used it in their practice to assess patient’s readiness to change their health behaviours. The expert panel reviewed all of the 36 items for literacy level and repeatedly modified the items based on redundancy, conciseness and clinical relevance. The panel came to a mutual agreement upon on a final set of 36 items that was pilot tested on a sample of patients with type 1 and type 2 diabetes at LMC clinics.

### Study population and procedures

Participants with a clinical diagnosis of type 1 or type 2 diabetes were recruited from seven southern Ontario LMC clinics. LMC clinics are multidisciplinary, regional community-based sites providing comprehensive care for patients with diabetes in Canada. A team approach is used incorporating Specialists, Physician Assistants, Registered Nurses, Dietitians and Pharmacists, using a common electronic medical record platform with shared care paths based on the Canadian Clinical Practice Guidelines [26]. Patients included in the current study had all been referred by their primary care physician and had been seeing healthcare providers at the LMC clinics for at least 6 months. All patients showed a persistent glycated hemoglobin level (HbA1c) ≥ 8.0% (64 mmol/mol) and had been triaged to a more comprehensive educational program (Advanced Self-Care Program). The study protocol and informed consent document were reviewed and approved by the research ethics board, IRB Services.

Data was collected from May 2014 to December 2014. The patients completed the SCPI in the LMC clinic with minimal assistance from staff, and the time to complete the questionnaire was recorded. Patients entered a score by producing a vertical mark on a Likert scale, which was then numerated. A score out of 10 was produced for the entire scale by adding up the score for each question and dividing by the total number of scale items (36). A score out of 10 was also produced for each subscale (Skills, Confidence and Preparedness) in a similar manner. Basic sociodemographic data were retrieved from patient records including age, gender, level of education, ethnicity, duration of diabetes, type of diabetes, date of diabetes diagnosis and HbA1c.

### Validity

Verification of the construct validity of the scale included investigating correlations with baseline socio-demographic and clinical variables, including age, gender, level of education, ethnicity, duration of diabetes, type of diabetes, and baseline HbA1c. To determine convergent validity, scale items were also correlated with two validated and commonly used diabetes assessment tools, The Diabetes Empowerment Scale (DES) [3] and the Michigan Diabetes Knowledge Test [9].

### Test re-test reliability

After patients first completed the SCPI, 70 patients completed it again in the LMC office approximately 6 months later. During these 6 months, patients took part in the Advanced Self-Care Program, where they received individualized diabetes education and care from the LMC healthcare team. The specific education that was provided to the participants was based on the gaps and barriers in patient self-care that were identified from the baseline completion of the SCPI.

### Data analysis

For detailed item analysis, it was determined that sample size of between 100 to 200 participants was required [31]. Item selection was informed by factor analysis, item-total statistics and the need for brevity. An exploratory factor analysis with a varimax rotation was performed. Items that had an Eigen value of greater than one and appeared before the elbow of the scree plot were retained [32]. Items with factor loadings less than 0.5 were removed from the scale. Subscales were built based on pre-defined groupings: skills, confidence and preparedness. The internal consistency of each subscale was estimated using Cronbach’s alpha score, leading to removal of items with a value less than 0.2 and items with a correlation > 0.8 [33]. Item-total correlation was used to remove items with a correlation of less than 0.2 [33, 34]. To assess construct validity of the scale, Pearson’s correlation coefficient was used for continuous variables, while Analysis of Variance (ANOVA) was used for categorical variables (gender, ethnicity and education). To investigate test-retest reliability, a sample of 70 participants who had received the test at baseline were administered the same test again 6 months later (after an interval education intervention). The Statistical Package for Social Sciences (SPSS) Version 20.0 (SPSS, Inc., 2009 Chicago, IL, USA) was used for analysis.

## Results

Thirty-six items were tested on 120 participants. Baseline characteristics of the participants are reported in Table 2. The mean age was 55.7 ± 12.7 years, half of the participants were male (51.7%), and the majority had type 2 diabetes (81.8%). The SCPI took an average of 10 min to complete. Readability of the SCPI was assessed with The Flesch-Kincaid Readability Test [35] producing a reading score of 63.9, consistent with standard English that is easily understood at an eighth or ninth grade level [35].

**Table 2:** Characteristics of participants

Variable	Statistic
Gender: n (%)	
Male	62 (51.7)
Female	58 (48.3)
Age: mean (SD)	55.77 (12.76)
Duration since diagnosis: mean (SD)^f^	14.91 (8.45)
Type of diabetes: n (%)^e^	
Type 1	20 (18.2)
Type 2	90 (81.8)
HbA1c: mean (SD)	9.47 (1.27)
Level of education: n (%)^a^	
Attended secondary school	17 (14.2)
Completed secondary school	21 (17.5)
College or technical diploma	20 (16.7)
Attended University	7 (5.8)
Completed University	21 (17.5)
Ethnicity: n (%)^b^	
Caucasian	71 (59.2)
South Asian	14 (11.7)
African	11 (9.2)
East/South East Asian	4 (3.3)
Caribbean	4 (3.3)
Other (Oceania, Arab, First Nations)	4 (3.3)
Average scores on tools: mean (SD)	
SCPI (min-max scores: 1-10)	6.5 (1.50)
MK (min-max scores: 0-100)	70 (17.3)
DE (min-max scores: 1-5)^c^	3.7 (0.68)
BTC (min-max scores: 0-14)^d^	8.5 (1.77)
PHQ9 (min-max scores: 1-27)	8.8 (7.44)

*SD* Standard deviation

^a^34 missing; ^b^7 missing; ^c^1 missing; ^d^ 6 missing; ^e^ 10 missing; ^f^ 13 missing

### Item selection

**Fig. 1:**
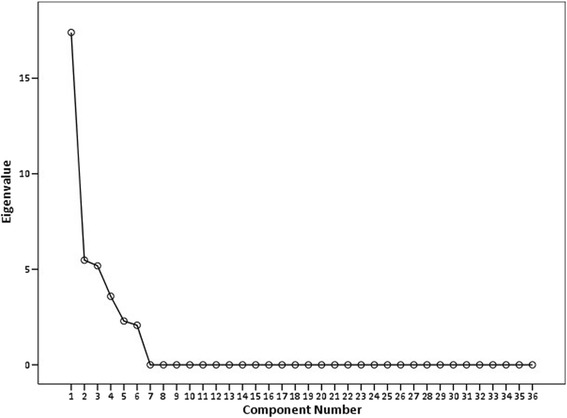
Scree plot showing elbow at six domains

The factor analysis revealed 6 components, which explained 100% of the total variance (Table 3 and Fig. 1). The component matrix is attached as Additional file 1. Two items with factor loadings of less than 0.5 were removed. The items total statistics identified three more items with item total correlations of less than 0.2. Six more items were deleted to further reduce the scale length, with no negative effect on the scale mean or on internal consistency. The item total statistics are reported in Additional file 2. In total, 11 items were removed, leaving us with 25 items with an overall intraclass correlation (ICC) of 0.94; 95% CI 0.92–0.99; *p* < 0.001. These items were divided into three subscales: Skills (9 items – questions 1, 2, 4, 5, 7, 8, 10, 12 and 22), Confidence (8 items – questions 3, 6, 11, 14, 16, 18, 19 and 21), Preparedness (8 items – questions 9, 13, 15, 17, 20, 23, 24 and 25). Subscale ICC’s ranged from 0.83 to 0.88. The full psychometric profile is reported in Table 4. The item selection process is outlined in Additional file 3 and the full 25-item SCPI can be found in Additional file 4.

**Table 3:** Total variance explained

	Initial Eigenvalues		
Component	Total	% of Variance	Cumulative %
1	17.395	48.319	48.319
2	5.478	15.216	63.535
3	5.180	14.390	77.924
4	3.587	9.963	87.887
5	2.291	6.364	94.251
6	2.070	5.749	100.000

**Table 4:** Psychometric properties of LMC SCPI index for 25 items

	Measure	Statistic
Reliability		
Internal consistency for subscales		
Skills	Cronbach’s alpha (95% CI)	0.85 (0.81- 0,89); 9 items
Confidence	Cronbach’s alpha (95% CI)	0.83 (0.78–0.87); 8 items
Preparedness	Cronbach’s alpha (95% CI)	0.88 (0.84–0.91); 8 items
Test-retest reliability	Pearson’s correlation coefficient; *p*-value	0.48; <0.001***
Validity		
Convergent validity		
Diabetes Empowerment^a^	Pearson’s correlation coefficient; *p*-value	0.28; 0.002**
Michigan Knowledge	Pearson’s correlation coefficient; *p*-value	0.21; 0.019*
PHQ9	Pearson’s correlation coefficient; *p*-value	-0.167; 0.068
Construct validity		
Age	Pearson’s correlation coefficient; *p*-value	-0.12; 0.173
Gender	ANOVA; F test, df; *p*-value	F[1, 118] = 0.018; 0.893
Level of education	ANOVA; F test, df; *p*-value	F[4,81] = 0.858;0.493
Ethnicity	ANOVA; F test, df; *p*-value	F[9,110] = 1.23; 0.286
Duration of diabetes	Pearson’s correlation coefficient; *p*-value	0.11; 0.256
Type of diabetes	ANOVA; F test, df; *p*-value	F [1, 108] = 5.433; 0.022*
A1C	Pearson’s correlation coefficient; *p*-value	-0.18;0.062; 104 participants

*p < 0.05. ** *p* < 0.01. *** *p* < 0.001

^a^1 missing

### Validity

The scores on the new scale (25 items) had a strong, positive correlation with the Diabetes Empowerment tool (*r* = 0.28;*p* = 0.002) and the Michigan Knowledge tool (*r* = 0.21; *p* = 0.019). Neither the socio-demographic variables assessed (age, gender, level of education, ethnicity, time since diagnosis) nor degree of depression (PHQ9) nor baseline HbA1c were significantly correlated with SCPI scores. Patients with Type 1 diabetes scored higher than patients with Type 2 diabetes (F [1, 108] = 5.433; *p* = 0.022; Table 4). We also noted that after 6 months, mean scores on the SCPI increased by 1.1; 95% CI 0.66–1.54; *p* < 0.001, and mean HbA1c was 1.3% (95% CI 0.92–1.69) lower compared to baseline HbA1c (*p* < 0.001).

### Test retest reliability

Test retest reliability was good (*r* = 0.48; *p* < 0.001) after 6 months in 70 participants (who had received interval education). There were no significant differences in baseline characteristics between the 120 participants who completed the SCPI at baseline and the sub-group of 70 participants who completed the SCPI at 6 months (*p* < 0.05).

## Discussion

This study presents the initial steps in evaluating the psychometric properties of a novel scale for measuring self-efficacy in people with diabetes. The SCPI is made up of three subscales: skills, confidence and preparedness, has excellent internal consistency, and compares favorably with other assessment tools. More importantly, it enables subsequent improvements in care via further specific education, training or other interventions. The length is practical (25 items compared to a median of 32 items in other scales), it is easy to read, and it can be self-administered in a clinical setting with minimal support from healthcare providers in a reasonable amount of time of 10 min.

The scale is independent of several socio-economic factors that were measured – specifically, age, gender, level of education, and ethnicity. Level of education has been an unfortunate confounding factor in current widely-used diabetes knowledge scales [9]. Further, the scale was independent of duration of diabetes and HbA1c.

Patients with type 1 diabetes scored higher overall than patients with type 2 diabetes, which may be due to the greater complexity of type 1 diabetes, earlier age at onset and the requisite foundational diabetes education and skills training. A similar pattern of higher knowledge scores [9] and higher quality of life scores [5] in type 1 diabetes has been observed in other diabetes indices.

It has been suggested that an instrument that measures self-care behaviours in patients with diabetes should be able to discriminate between patients with good versus poor glycemic control [19]. Although the association between baseline HbA1c and SCPI scores was not statistically significant in the present study, we may have been underpowered for this association, as only 104 participants had available data for HbA1c. As well, all patients in this particular cohort were in a category of poor glycemic control as per the HbA1c >8.0% inclusion criteria. Thus, the association between HbA1c and SCPI scores should be evaluated in a larger sample, with a broader range of HbA1c values.

A commonly used tool, the Diabetes Empowerment Scale, [3] arose out of research in empowerment-training and intentionally avoided inquiries specific to individual behaviours. The authors preferred creation of a tool to measure the impact of an educational intervention and accepted the resulting limitation in assessment of specific behaviours. In contrast, development of the LMC Skills, Confidence & Preparedness Index (SCPI) was inspired by a HCPs lacking measurable insight into specific behaviours. It was therefore intentionally derived from commonly identified deficits in patient knowledge and recurring gaps in either confidence, or in patient preparedness even when confidence is high. These deficits and gaps are further commonly linked to specific behavioural themes, each integral to optimal diabetes self-care. We believe that the comprehensive and specific nature of the SCPI item sources - healthy eating, being active, blood glucose monitoring, taking medication, reducing risk, problem solving and healthy coping - adds further value to the healthcare provider using the tool. Although other scales have been created to specifically assess diabetes self-care, [21] self-efficacy of diabetes care, [3, 12] knowledge of diabetes, [9, 11] and confidence in diabetes self-care, [22], SCPI is the first scale to simultaneously evaluate skills, confidence and preparedness of diabetes self-management. Gaps or deficits illustrated in the SCPI responses can then directly lead to an appropriate care path response.

Although a variety of diabetes assessment tools are available, some issues may limit their applicability. Many of the questionnaires used in practice today are >10 years old, [3, 9, 21] and thus do not reflect the updated therapies available and current diabetes care standards for patients living with diabetes [36]. Since their development, more injectable non-insulin therapies have become available, often used in combination with insulin and oral therapies, contributing to a significant increase in complexity of self-care, which may not be fully explored within prior tools. Even dietary management, in which ‘carbohydrate counting’ has become ubiquitous in guiding self-care decisions, requires a depth of inquiry which was not apparent when prior tools were developed. Secondly, there is limited clinical relevance to many questionnaires currently used in practice for healthcare providers because their original development mission was to measure broad outcomes of an intervention, [20] rather than to develop insights into specific behaviours and then guide specific interventions. Finally, although other assessment tools are widely used to assess “patient activation”, [37, 38] they are not specific to the self-efficacy of diabetes management. Accordingly, the SCPI offers several advantages compared to other available diabetes assessment tools. The SCPI was specifically designed to allow for easy administration in a busy clinical setting. Patients were able to complete the SCPI with minimal assistance from staff, in a timely manner of only 10 min, generally suggested to be the maximum time to complete a health questionnaire [36] Furthermore, the comprehensive nature of the SCPI allows health care professionals to determine which domains of skills, confidence and preparedness the patient may be struggling with, thus allowing for more individualized care that targets the specific needs of patients.

Several issues warrant further discussion. Even though the factor analysis identified six domains which explained 100% of the variance in the scale, we had intentionally pre-selected three domains: skills, confidence and preparedness. We did, however, confirm that the internal consistency of these domains was also very good, with Cronbach’s coefficient alpha for all sub-scales exceeding the recommended 0.70 for new scale development [39]. Secondly, we have not strictly evaluated test- retest reliability per se, but rather the sensitivity of the scale to diabetes education. After 6 months, participants scored significantly higher on the SCPI, and also had significantly improved HbA1c. This finding might lead to the hypothesis that interventions designed to address SCPI-identified gaps in patient self-management may lead to successful lowering of HbA1c. Further, our results suggest that the SCPI can be used to evaluate improvement in skills, confidence and preparedness of diabetes self-management following an educational intervention. Therefore, the SCPI shows evidence of responsiveness to change, and ability to reflect intervention effects, an important characteristic for health measurement tools [36]. However, due to the education intervention and the 6-month interval, further investigation is needed to strictly verify test-retest reliability of the SCPI. Further, although scores increased following a diabetes education intervention, the minimal important change (MIC) of SCPI scores [40] is yet to be determined and warrants further investigation. Finally, the study was conducted in tertiary referral centres, in the context of a special patient education program in self-care, and for hyperglycemic patients (HbA1c ≥ 8% [64 mmol/mol]), possibly introducing a selection bias. We are currently evaluating the reliability and validity of the SCPI using an electronic platform in patients with a broader range of HbA1c values, who are not undergoing formal diabetes education.

## Conclusion

In conclusion, this pilot study indicates that the SCPI is a valid and reliable instrument to assess self-management behaviours in patients with diabetes, and presents evidence to inform further testing and validation of the SCPI. The Skills, Confidence & Preparedness Index (SCPI) represents the first patient assessment tool designed to evaluate each of these three attributes independently, in a brief questionnaire, which simultaneously provides guidance for subsequent intervention for healthcare providers in diabetes. The tool shows excellent validity and reliability, correlates well with existing tools and is not affected by most sociodemographic differences in our test population, including prior level of education. The initial mean increase in score, following a 6 month intervention period, in conjunction with improved HbA1c, suggests that it may also reliably measure response to an intervention and that it may correlate with clinically meaningful outcomes. Additional research is needed to further assess the validity and reliability of the SCPI in broader diabetes patient populations to continue to assess its potential contribution. The SCPI may be used to assess self-management behaviours in patients with diabetes, to determine educational needs and to support clinical management.

## Additional files

Additional file 1: Component Matrix. The factor loadings of each scale item on all 6 components identified by the factor analysis. (DOCX 16 kb)
Additional file 2: Item-total statistics. Item statistics if each SCPI item were deleted. (DOCX 16 kb)
Additional file 3: Selection Process. A table showing which items were deleted at different stages of scale development and analysis (DOCX 18 kb)
Additional file 4: LMC Diabetes Skills, Confidence & Preparedness Index (SCPI). The final 25-item SCPI (DOCX 75 kb)

## Abbreviations

AADE: American Association of Diabetes Educators
ANOVA: Analysis of variance
CPG: Clinical Practice Guidelines
DES: Diabetes Empowerment Scale
HbA1c: Hemoglobin A1c
HCP: Health care professional
ICC: Intraclass correlation coefficient
MIC: Minimal important change
PHQ9: Patient Health Questionnaire
r: Pearson Product Moment Correlation Coefficient
SCPI: Skills, Confidence & Preparedness Index
