# Supplementary material for: The LMC Skills, Confidence & Preparedness Index (SCPI): development and evaluation of a novel tool for assessing self-management in patients with diabetes
Source: Health Qual Life Outcomes. 2017 Jan 31;15:27. doi: 10.1186/s12955-017-0606-z (PMC5282708; doi:10.1186/s12955-017-0606-z)
Supplement: Additional file 1: — Component Matrix. The factor loadings of each scale item on all 6 components identified by the factor analysis. (DOCX 16 kb) [file 12955_2017_606_MOESM1_ESM.docx]

**Additional file 1: Component Matrix**

| **Items** | **Component** | | | | | |
| --- | --- | --- | --- | --- | --- | --- |
|  | **1** | **2** | **3** | **4** | **5** | **6** |
| SCPI11 | .618 | -.371 | -.342 | .536 | .121 | -.248 |
| SCPI12 | -.277 | -.304 | -.510 | .679 | -.329 | .030 |
| SCPI13 | .388 | -.837 | .127 | -.120 | -.334 | .090 |
| SCPI14 | .724 | -.595 | .184 | -.047 | .091 | -.281 |
| SCPI15 | .130 | .781 | .480 | -.225 | -.093 | .288 |
| SCPI16 | .276 | .497 | .175 | .254 | .555 | .524 |
| SCPI17 | .575 | .322 | -.607 | .423 | .100 | .085 |
| SCPI18 | .752 | .172 | -.573 | .094 | -.231 | .122 |
| SCPI19 | .902 | .079 | -.204 | -.277 | -.231 | .086 |
| SCPI110 | .392 | .181 | .572 | .628 | -.131 | .274 |
| SCPI111 | .928 | .114 | .224 | .182 | -.160 | -.127 |
| SCPI112 | .874 | -.240 | .201 | .035 | .370 | -.009 |
| SCPI113 | .842 | -.398 | -.218 | -.267 | -.022 | .114 |
| SCPI114 | .599 | -.247 | -.503 | .275 | .500 | .039 |
| SCPI115 | .762 | -.469 | .244 | .048 | .370 | -.003 |
| SCPI116 | .735 | .311 | .033 | -.497 | -.233 | .247 |
| SCPI117 | .243 | -.257 | .698 | .598 | -.083 | .152 |
| SCPI118 | .906 | .267 | .052 | .101 | -.261 | -.164 |
| SCPI119 | .991 | -.070 | .096 | -.041 | .049 | .001 |
| SCPI120 | .650 | -.568 | .389 | .186 | .188 | .185 |
| SCPI121 | .833 | .499 | -.164 | .153 | -.045 | .073 |
| SCPI122 | .190 | -.126 | -.788 | .265 | .077 | .500 |
| SCPI123 | .352 | .784 | .141 | .004 | .456 | -.181 |
| SCPI124 | -.500 | .306 | .712 | .179 | .225 | .259 |
| SCPI125 | .851 | .420 | -.036 | .121 | -.281 | .074 |
| SCPI126 | .915 | .078 | .348 | .068 | .114 | -.132 |
| SCPI127 | .901 | -.083 | .252 | -.239 | -.233 | .076 |
| SCPI128 | .757 | -.179 | -.045 | -.496 | .319 | -.212 |
| SCPI129 | .820 | -.374 | -.026 | -.198 | .174 | .343 |
| SCPI130 | .882 | .187 | -.011 | -.288 | .221 | -.235 |
| SCPI131 | .894 | .135 | -.196 | .044 | -.104 | -.363 |
| SCPI132 | .486 | .561 | .286 | .272 | -.259 | -.475 |
| SCPI133 | .401 | -.289 | .575 | .599 | -.240 | -.098 |
| SCPI134 | .961 | .239 | .043 | -.122 | -.044 | -.025 |
| SCPI135 | .581 | -.239 | .162 | -.360 | -.384 | .550 |
| SCPI136 | .496 | .440 | -.695 | .252 | -.053 | .105 |
| Extraction Method: Principal Component Analysis. | | | | | | |
| a. 6 components extracted. | | | | | | |
