# Supplementary material for: The LMC Skills, Confidence & Preparedness Index (SCPI): development and evaluation of a novel tool for assessing self-management in patients with diabetes
Source: Health Qual Life Outcomes. 2017 Jan 31;15:27. doi: 10.1186/s12955-017-0606-z (PMC5282708; doi:10.1186/s12955-017-0606-z)
Supplement: Additional file 2: — Item-total statistics. Item statistics if each SCPI item were deleted. (DOCX 16 kb) [file 12955_2017_606_MOESM2_ESM.docx]

**Additional file 2: Item total statistics**

| **Items** | **Scale Mean if Item Deleted** | **Scale Variance if Item Deleted** | **Corrected Item-Total Correlation** | **Cronbach's Alpha if Item Deleted** |
| --- | --- | --- | --- | --- |
| SCPI11 | 231.71 | 3091.905 | .629 | .952 |
| SCPI12 | 229.86 | 3375.476 | -.248 | .959 |
| SCPI13 | 229.57 | 3224.952 | .311 | .954 |
| SCPI14 | 230.00 | 3115.333 | .655 | .952 |
| SCPI15 | 230.14 | 3281.810 | .111 | .955 |
| SCPI16 | 233.57 | 3209.952 | .311 | .954 |
| SCPI17 | 231.29 | 3121.238 | .617 | .952 |
| SCPI18 | 230.86 | 3063.143 | .752 | .951 |
| SCPI19 | 231.29 | 2958.571 | .852 | .950 |
| SCPI110 | 229.57 | 3169.619 | .436 | .954 |
| SCPI111 | 231.14 | 2940.476 | .926 | .950 |
| SCPI112 | 228.14 | 3131.810 | .857 | .952 |
| SCPI113 | 231.00 | 3038.667 | .780 | .951 |
| SCPI114 | 230.14 | 3073.143 | .597 | .953 |
| SCPI115 | 229.43 | 3053.286 | .724 | .951 |
| SCPI116 | 230.43 | 3162.619 | .671 | .952 |
| SCPI117 | 229.14 | 3261.810 | .282 | .954 |
| SCPI118 | 230.57 | 2996.286 | .896 | .950 |
| SCPI119 | 230.14 | 2991.810 | .972 | .950 |
| SCPI120 | 229.57 | 3086.952 | .624 | .952 |
| SCPI121 | 230.57 | 3082.619 | .854 | .951 |
| SCPI122 | 229.86 | 3233.810 | .210 | .955 |
| SCPI123 | 230.43 | 3195.619 | .329 | .954 |
| SCPI124 | 229.57 | 3393.952 | -.482 | .958 |
| SCPI125 | 229.71 | 3027.905 | .858 | .950 |
| SCPI126 | 229.43 | 3083.286 | .902 | .951 |
| SCPI127 | 231.00 | 3099.333 | .850 | .951 |
| SCPI128 | 229.29 | 3096.238 | .655 | .952 |
| SCPI129 | 227.71 | 3202.905 | .790 | .953 |
| SCPI130 | 229.29 | 2927.905 | .815 | .951 |
| SCPI131 | 229.00 | 3138.667 | .873 | .952 |
| SCPI132 | 229.86 | 3161.810 | .468 | .953 |
| SCPI133 | 228.71 | 3203.905 | .416 | .954 |
| SCPI134 | 229.86 | 2957.810 | .937 | .950 |
| SCPI135 | 228.86 | 3142.143 | .511 | .953 |
| SCPI136 | 229.29 | 3130.238 | .513 | .953 |
