# Supplementary material for: The LMC Skills, Confidence & Preparedness Index (SCPI): development and evaluation of a novel tool for assessing self-management in patients with diabetes
Source: Health Qual Life Outcomes. 2017 Jan 31;15:27. doi: 10.1186/s12955-017-0606-z (PMC5282708; doi:10.1186/s12955-017-0606-z)
Supplement: Additional file 3: — Selection Process. A table showing which items were deleted at different stages of scale development and analysis (DOCX 18 kb) [file 12955_2017_606_MOESM3_ESM.docx]

**Additional file 3: Item selection process**

| **Items derived from expert deliberation** | **Dropped after Factor Analysis** | **Dropped after Item-Total statistics** | **Dropped based on Cronbach’s alpha** |
| --- | --- | --- | --- |
| 1. I am able to portion out and choose foods that have the optimal balance between carbohydrates, proteins and vegetables to help keep my blood sugars in target. |  |  |  |
| 1. I plan to start checking my feet on a daily basis and booking all of my necessary diabetes appointments in the coming week. |  |  |  |
| 1. I am confident that I can regularly look at my meter or logbook to figure out patterns. |  |  |  |
| 1. I know how my diabetes insulin or medication works in my body and at which time of day I should check my blood sugars to make sure my dose is correct. |  |  |  |
| 1. When I am out of the house in the next week, I will choose foods that help keep my blood sugars in target. |  |  |  |
| 1. I know the three different types of exercise and their benefits on my health and diabetes. |  |  |  |
| 1. I feel confident that I can plan balanced meals and snacks effectively. |  |  |  |
| 1. If I miss a dose of my insulin or medication, I know how my body will react and the steps to take to get back on track. |  |  |  |
| 1. When I am planning to exercise, I know what changes I need to make to avoid a low blood sugar before, during, and after exercise. |  |  |  |
| 1. I know **all** the parts of my body that can be affected by high blood sugars, what happens to those parts if I don’t take good care of my blood sugars and what I should do to prevent, monitor and treat the complications. |  |  |  |
| 1. I am confident that I can implement stress management techniques into my lifestyle. |  |  |  |
| 1. I know when to check my blood sugar if I want to see how my body reacted to a meal. |  |  |  |
| 1. When I am sitting at a restaurant, I can identify the best choices on the menu that would help keep my blood sugars in target. |  |  |  |
| 1. If I get sick, I am confident that I will make the necessary changes to my medications, insulin and/or eating to manage my blood sugars. |  |  |  |
| 1. When I am sick, I know what to do differently with my medications, fluid intake, food intake, blood sugar testing, and when to go to the hospital. |  |  |  |
| 1. I intend to start planning and eating balanced meals and snacks starting next week. |  |  |  |
| 1. I am confident that I can keep a record of my own ‘ABC’ results and that I know what my targets. |  |  |  |
| 1. I know how to identify stress in my life and how it can impact my diabetes management & overall health. |  |  |  |
| 1. I’m confident that I can plan ahead for what to do, and how to react, either before, during or after exercise to avoid a low blood sugar. |  |  |  |
| 1. When I look at my blood sugars in my meter or in my logbook in a given week, I could explain to my diabetes educator or doctor what my blood sugar pattern is. |  |  |  |
| 1. I plan to choose an activity and begin incorporating it into my schedule in the coming week. |  |  |  |
| 1. In the next week, I will create a plan on how to remember to take my medications and on what to do if I miss my medications. |  |  |  |
| 1. I am confident that at the next time I am eating out of my home, I will be able to plan and select the foods that best keep my blood sugars under control. |  |  |  |
| 1. I plan to keep a record of my own ‘ABC’ results in the next week and ask my healthcare team for help if my results are not meeting their appropriate target. |  |  |  |
| 1. I plan to start using my blood sugar levels to make changes to my diet and/or insulin starting next week. |  |  |  |
| 1. I am confident that I can choose a healthy activity for me and include it into my schedule. |  |  |  |
| 1. I plan to start making a list of stress management techniques which will work for me in the upcoming week. |  |  |  |
| 1. I am confident that I can adjust my insulin or medication doses, on my own, to reach the target blood sugar levels. |  |  |  |
| 1. I am confident that I can commit to preventing and monitoring my diabetes complications such as seeing my eye doctor at least once a year and checking my feet on a daily basis. |  |  |  |
| 1. I plan to start adjusting my insulin or medication doses on my own starting next week. |  |  |  |
| 1. I am confident that I will use my blood sugar results to make changes to my diet and/or insulin to help keep my blood sugars in target. |  |  |  |
| 1. I know what the ABCs (A1c, Blood Pressure, and Cholesterol) of Diabetes are, what my targets are and how they impact my diabetes. |  |  |  |
| 1. I plan to start looking for patterns in my meter or logbook starting next week. |  |  |  |
| 1. The next time I am sick, I will make the necessary changes to my medications, insulin and/or eating depending on my blood sugars. |  |  |  |
| 1. With my next exercise, I am going to make a plan to reduce the chance of a low blood sugar, or to react with a good response if I do have a low blood sugar. |  |  |  |
| 1. I am confident that I can plan ahead to avoid missing my medications and that if I did miss a medication or insulin, I would know what to do. |  |  |  |
