# Supplementary material for: The LMC Skills, Confidence & Preparedness Index (SCPI): development and evaluation of a novel tool for assessing self-management in patients with diabetes
Source: Health Qual Life Outcomes. 2017 Jan 31;15:27. doi: 10.1186/s12955-017-0606-z (PMC5282708; doi:10.1186/s12955-017-0606-z)
Supplement: Additional file 4: — LMC Diabetes Skills, Confidence & Preparedness Index (SCPI). The final 25-item SCPI (DOCX 75 kb) [file 12955_2017_606_MOESM4_ESM.docx]

LMC Diabetes Skills, Confidence & Preparedness Index (SCPI)

Answer the following questions on a scale of 1-10 (1= very little and 10= a lot). Please do this by drawing a line on the scale where you see yourself for each question.

1. I am able to portion out and choose foods that have the optimal balance between carbohydrates, proteins and vegetables to help keep my blood sugars in target.

1 (very little) 10 (a lot)

1. I know how my diabetes insulin or medication works in my body and at which time of day I should check my blood sugars to make sure my dose is correct.

1 (very little) 10 (a lot)

1. I feel confident that I can plan balanced meals and snacks effectively.

1 (very little) 10 (a lot)

1. If I miss a dose of my insulin or medication, I know how my body will react and the steps to take to get back on track.

1 (very little) 10 (a lot)

1. When I am planning to exercise, I know what changes I need to make to avoid a low blood sugar before, during, and after exercise.

1 (very little) 10 (a lot)

1. I am confident that I can implement stress management techniques into my lifestyle.

1 (very little) 10 (a lot)

1. I know when to check my blood sugar if I want to see how my body reacted to a meal.

1 (very little) 10 (a lot)

1. When I am sick, I know what to do differently with my medications, fluid intake, food intake, blood sugar testing, and when to go to the hospital.

1 (very little) 10 (a lot)

1. I intend to start planning and eating balanced meals and snacks starting next week.

1 (very little) 10 (a lot)

1. I know how to identify stress in my life and how it can impact my diabetes management & overall health.

1 (very little) 10 (a lot)

1. I’m confident that I can plan ahead for what to do, and how to react, either before, during or after exercise to avoid a low blood sugar.

1 (very little) 10 (a lot)

1. When I look at my blood sugars in my meter or in my logbook in a given week, I could explain to my diabetes educator or doctor what my blood sugar pattern is.

1 (very little) 10 (a lot)

1. I plan to choose an activity and begin incorporating it into my schedule in the coming week.

1 (very little) 10 (a lot)

1. I am confident that at the next time I am eating out of my home, I will be able to plan and select the foods that best keep my blood sugars under control.

1 (very little) 10 (a lot)

1. I plan to start using my blood sugar levels to make changes to my diet and/or insulin starting next week.

1 (very little) 10 (a lot)

1. I am confident that I can choose a healthy activity for me and include it into my schedule.

1 (very little) 10 (a lot)

1. I plan to start making a list of stress management techniques which will work for me in the upcoming week.

1 (very little) 10 (a lot)

1. I am confident that I can adjust my insulin or medication doses, on my own, to reach the target blood sugar levels.

1 (very little) 10 (a lot)

1. I am confident that I can commit to preventing and monitoring my diabetes complications such as seeing my eye doctor at least once a year and checking my feet on a daily basis.

1 (very little) 10 (a lot)

1. I plan to start adjusting my insulin or medication doses on my own starting next week.

1 (very little) 10 (a lot)

1. I am confident that I will use my blood sugar results to make changes to my diet and/or insulin to help keep my blood sugars in target.

1 (very little) 10 (a lot)

1. I know what the ABCs (A1c, Blood Pressure, and Cholesterol) of Diabetes are, what my targets are and how they impact my diabetes.

1 (very little) 10 (a lot)

1. I plan to start looking for patterns in my meter or logbook starting next week.

1 (very little) 10 (a lot)

1. The next time I am sick, I will make the necessary changes to my medications, insulin and/or eating depending on my blood sugars.

1 (very little) 10 (a lot)

1. With my next exercise, I am going to make a plan to reduce the chance of a low blood sugar, or to react with a good response if I do have a low blood sugar.

1 (very little) 10 (a lot)
